# Supplementary material for: A retrospective two centre study of Birt-Hogg-Dubé syndrome reveals a pathogenic founder mutation in FLCN in the Swedish population
Source: PLoS One. 2022 Feb 17;17(2):e0264056. doi: 10.1371/journal.pone.0264056 (PMC8853502; doi:10.1371/journal.pone.0264056)
Supplement: Attachment — Submitted filename: Response to Reviewers.docx [file pone.0264056.s003.docx]

A retrospective two centre study of Birt-Hogg-Dubé syndrome reveals a pathogenic founder mutation in FLCN in the Swedish population

Response to reviewer comments, each response marked in bold.

**REVIEWER 1**

1. Authors could add the sampling location: with a map of Sweden, locate geographic origins of families from where the samples were collected? **All patients reside from the same location, Stockholm-Uppsala region. This has been changed in the text page 4, line 70-71.**
2. Authors could also determine the age of founder effect mutation in Swedish population, taking generation time as 30 years? **Determination of the age of founder effect mutation was not within the scope of our study and therefore we did not calculate it.**
3. Since these mutations are present in the family, it is worth checking whether these are linked in family? **We do not fully understand the particular question. Is it a LOD calculation that reviewer is asking here? If so, this is meant for Linkage analysis studies and this wasn’t either within the scope of our work.**
4. Talking about mutations, did authors also considered heterozygotes or only considered homozygotes in their analysis? **Birt-Hogg-Dube syndrome is an autosomal dominant syndrome, hence heterozygous variants were evaluated.** **Homozygous loss of FLCN causes embryonic lethality in mice (Hasumi et al., Homozygous loss of BHD causes early embryonic lethality with activation of mTORC1 and mTORC2. PNAS (2009, Nov 3; 106(44): 18722-18727).**

   Minor comments:
5. Some sentences need to be checked again like “Single nucleotide variants were called using Mutation Identification Pipeline (MIP) (ref GitHub).” Please provide Github reference. **Reference has added in page 6, line 100.**
6. Figure resolutions are poor and should increase. **The resolution has been increased to 300 dpi.**

**REVIEWER 2**

1. Did the authors find any mutations from non-coding and yet those coming from 5' UTRs/enhancers? **We only sequenced coding regions (exones), this has been clarified in the manuscript, page 5, line 90-91.**
2. Any variants of unknown significance? **We reported only pathogenic and likely pathogenic variants in our work.**
3. There must be a methodological flowchart which would be a very nice addition. **Samples were first Sanger sequenced or analyzed using a multigene panel which included *FLCN,* and if no variant was detected they were then analyzed with MLPA to identify gains or losses in the *FLCN* gene. We did not provide a methodological flowchart to visualize this. It has been clarified in the text, page 5, line 86-88.**
4. The figures must be of high resolution. **The resolution has been increased to 300 dpi.**
5. A work of such magnitude would be of great deal if this could be associated with a development of panel, for example n360. **A panel has not been developed; it was not within the scope of the study. However, we would like to delineate that the FLCN gene is included in a vast majority of cancer panels worldwide already, which implies that no further technological development is needed concerning the diagnostic aspect for the syndrome for the variants we report. On the contrary, by updating the literature with our findings, especially regarding the “Swedish founder mutation” which is reported only in a handful of patients, clinics and laboratories around the globe which are working with these cancer panels will benefit from keep their products (panels) up-to-date, in particular for patients with Swedish origin.**
6. The authors could describe the applications of their study towards precision medicine. **In the discussion we do stress the importance of developing international guidelines for Birt-Hogg-Dube syndrome regarding diagnosis and management of the disorder, page 19 line 322-327.**

The authors thank reviewers for their valuable comments!
