# Supplementary material for: A retrospective two centre study of Birt-Hogg-Dubé syndrome reveals a pathogenic founder mutation in FLCN in the Swedish population
Source: PLoS One. 2022 Feb 17;17(2):e0264056. doi: 10.1371/journal.pone.0264056 (PMC8853502; doi:10.1371/journal.pone.0264056)
Supplement: Attachment — Submitted filename: Response to Reviewers_v2.docx [file pone.0264056.s004.docx]

A retrospective two center study of Birt-Hogg-Dubé syndrome reveals a pathogenic founder mutation in FLCN in the Swedish population

Response to reviewer comments, each response marked in bold. Additional comments are marked in blue.

**REVIEWER 1**

1. Authors could add the sampling location: with a map of Sweden, locate geographic origins of families from where the samples were collected? **All patients reside from the same location, Stockholm-Uppsala region. This has been changed in the text page 4, line 70-71.**

For further information these two regions (Stockholm and Uppsala) are in the middle part of Sweden and the two regions provides clinical genetic services to approximately 1/3 of the population in Sweden.

2. Authors could also determine the age of founder effect mutation in Swedish population, taking generation time as 30 years? **Determination of the age of founder effect mutation was not within the scope of our study and therefore we did not calculate it.**

Additional information: in order to make a good determination regarding age of the founder mutation allele frequencies for the used microsatellites/markers has to be known. Unfortunately, we do not have access to a substantial number of individuals representing the whole of the Swedish population to determine these frequencies. So unfortunately, this is not within the scope of our study.

3. Since these mutations are present in the family, it is worth checking whether these are linked in family? **We do not fully understand the particular question. Is it a LOD calculation that reviewer is asking here? If so, this is meant for Linkage analysis studies and this wasn’t either within the scope of our work.**

Additional information: The paper describes the phenotype and genotype of probands and relatives in the families. As can be read in the tables genotype and phenotype are linked in the families. If this needs to be further emphasized please inform us.

4. Talking about mutations, did authors also considered heterozygotes or only considered homozygotes in their analysis? **Birt-Hogg-Dube syndrome is an autosomal dominant syndrome, hence heterozygous variants were evaluated.** **Homozygous loss of FLCN causes embryonic lethality in mice (Hasumi et al., Homozygous loss of BHD causes early embryonic lethality with activation of mTORC1 and mTORC2. PNAS (2009, Nov 3; 106(44): 18722-18727).**

Additional information: We only considered heterozygous variants – homozygous variants were not detected.

Minor comments:

5. Some sentences need to be checked again like “Single nucleotide variants were called using Mutation Identification Pipeline (MIP) (ref GitHub).” Please provide Github reference. **Reference has added in page 6, line 101.**
6. Figure resolutions are poor and should increase. **The resolution has been increased to 300 dpi.**

Additional information: The figures now have the resolution that is required from the journal.

**REVIEWER 2**

1. Did the authors find any mutations from non-coding and yet those coming from 5' UTRs/enhancers? **We only sequenced coding regions (exones), this has been clarified in the manuscript, page 5, line 91-92.**

Clarification – the potential UTRs and enhancers were not analyzed. These regions are almost never included in a clinical diagnostic testing since the scientific knowledge regarding these regions still are poor – especially for inclusion in clinical genetic testing.

2. Any variants of unknown significance? **We reported only pathogenic and likely pathogenic variants in our work.**

Addition info: Variants of unknown clinical significance are rarely found in the FLCN gene – and if we find it we are almost always able to do segregation analysis in affected families, therefor the classification changes to possibly benign or likely pathogenic in regards to the ACMG criteria for classification of reported variants.

3. There must be a methodological flowchart which would be a very nice addition. **Samples were first Sanger sequenced or analyzed using a multigene panel which included *FLCN,* and if no variant was detected they were then analyzed with MLPA to identify gains or losses in the *FLCN* gene. We did not provide a methodological flowchart to visualize this. It has been clarified in the text, page 5, line 87-89.**

Clarification – the flowchart would be very simple and can be included as an additional file (supplementary material) if requested. See below how this could be illustrated, but we hope that the clarification in the manuscript has solved this.

4. The figures must be of high resolution. **The resolution has been increased to 300 dpi.**

Additional information: The figures now have the resolution that is required from the journal.

5. A work of such magnitude would be of great deal if this could be associated with a development of panel, for example n360. **A panel has not been developed; it was not within the scope of the study. However, we would like to delineate that the FLCN gene is included in a vast majority of cancer panels worldwide already, which implies that no further technological development is needed concerning the diagnostic aspect for the syndrome for the variants we report. On the contrary, by updating the literature with our findings, especially regarding the “Swedish founder mutation” which is reported only in a handful of patients, clinics and laboratories around the globe which are working with these cancer panels will benefit from keep their products (panels) up-to-date, in particular for patients with Swedish origin.**

Additional info: Birt-Hogg-Dube is a monogenic autosomal dominant disorder with a high rate of detecting pathogenic variants in affected individuals using sequencing and MLPA (>96% of patients will have a genetic diagnosis after test). No other gene is so far known to be affected in Birt-Hogg-Dube syndrome.

6. The authors could describe the applications of their study towards precision medicine. **In the discussion we do stress the importance of developing international guidelines for Birt-Hogg-Dube syndrome regarding diagnosis and management of the disorder, page 19 line 321-328.**

Additional info: We sincerely hope that the finding of a founder mutation and the description of the range of phenotypes individuals with the same disease-causing variant – as described in this manuscript – will increase the knowledge regarding precision medicine towards Birt-Hogg-Dube syndrome.

The authors thank reviewers for their valuable comments!

Sanger sequencing

Pathogentic variant detected

Yes

No

MLPA

Report

Report
